# Unveiling the dark side of *Prevotella*: a case of fatal pneumonia from a common probiotic

**DOI:** 10.3389/fmed.2024.1382134

**Published:** 2024-12-24

**Authors:** Meng-Jie Li, Shou-Feng Zhou, Yu Zhang, Yong Zhang, Wen-Bo Fu

**Affiliations:** ^1^Department of Respiratory Oncology, Renmin Hospital of Qingxian, Cangzhou, China; ^2^Department of Digestive Diseases, General Hospital of Central Theater Command of the People’s Liberation Army, Wuhan, China; ^3^Department of Pathology, General Hospital of Central Theater Command of the Chinese People’s Liberation Army, Wuhan, China; ^4^Department of Integrative Medicine, General Hospital of Central Theater Command of the People’s Liberation Army, Wuhan, China; ^5^Department of Cardiology, General Hospital of Central Theater Command of the People’s Liberation Army, Wuhan, China

**Keywords:** *Prevotella*, gum pain, septic emboli, necrotizing mediastinitis, Lemierre’s syndrome

## Abstract

*Prevotella* is often considered a *Bacteroides* complex associated with a healthy plant-based diet that acts as a “probiotic” throughout the body’s entire digestive tract from the mouth to the anus. Previous studies have not reported that this “probiotic” colonizing the human body could cause severe pneumonia. This case report describes a 56-year-old healthy female worker with gum pain followed by fever. Despite prompt medical attention given by the use of empirical antibiotics and tooth and oral cleaning, the disease rapidly progressed to retropharyngeal abscess and severe pneumonia. Although the surgeon performed pharynx and cervical incisions and drainage, the patient’s symptoms were not significantly relieved. After repeated blood culture and sputum culture, no positive findings were found. Fortunately, *Prevotella oris* was found in the peripheral blood of the patient by metagenomic next-generation sequencing (mNGS). The disease was controlled quickly by changing the targeted antibiotics according to the guidelines for the detection of pathogenic bacteria. Three months after discharge, the patient’s symptoms did not resolve, and reexamination with computerized tomography (CT) showed that the neck and chest were normal. This case is unique in that it shows that normally colonized *Prevotella oris* could also cause fatal pneumonia as an opportunistic pathogen. Our goal is to highlight that serious infections that rapidly develop from common symptoms in an era of widespread antibiotic use not only increase patient misunderstanding but also lead to over detection and testing of such symptoms by clinicians. Expanding the pathogenic characteristics of special pathogens through the literature and using accurate mNGS may be the technical tool for resolving this contradiction.

## Introduction

*Prevotella* is often considered a *Bacteroides* complex associated with a healthy plant-based diet that acts as a “probiotic” throughout the body’s entire digestive tract from the mouth to the anus (1, 2). Previous studies have not reported that this “probiotic” colonizing the human body could cause oropharyngeal infections to severe pneumonia. Oropharyngeal infections resulting from odontogenic, pharyngeal, or other causes can spread through multiple spaces through the three cervical fascial planes of the external, middle and deep layers (3, 4). Oropharyngeal infections may cause severe infections with potentially life-threatening complications, such as Lemierre’s syndrome, descending necrotizing mediastinitis, suppurative thrombophlebitis of the internal jugular vein associated with pulmonary septic embolism, thrombosis of the cavernous sinus and erosion of the carotid artery (5, 6). Oropharyngeal infections are usually polymicrobial in nature and include *Streptococci*, *Fusobacterium necrophorum*, *Bacteroides*, *Peptostreptococcus species*, *Staphylococcus aureus*, and *anaerobes* (7, 8). The oral microbiome is one of the most diverse microbiomes in the human body, with more than 700 species identified (9). This case report is the first to show that a normally colonized oral *Prevotella* pathogen acts as an opportunistic pathogen, progressing rapidly from common gum pain and fever to oropharyngeal infections and severe pneumonia, from empirical treatment to precision medicine.

## Case report

**FIGURE 1:**
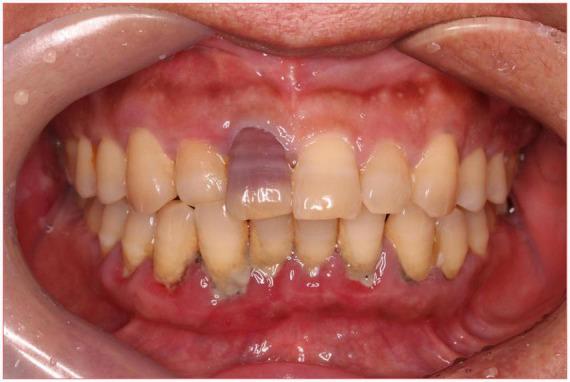
Oral examination revealed that the patient’s gums were red and swollen, and several teeth had heavy stones.

**FIGURE 2:**
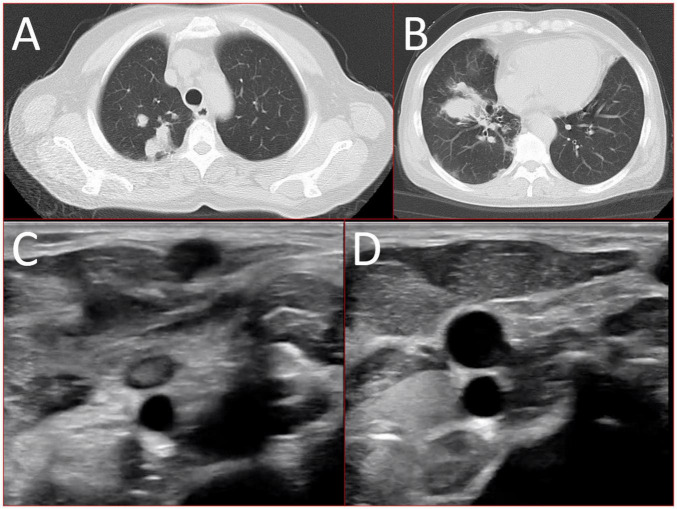
16-slice computerized tomography images of the chest at initial diagnosis. **(A)** Upper lobe; **(B)** lower lobe) and ultrasound images of the neck at initial diagnosis **(C)** inflammatory changes; **(D)** local effusion.

**FIGURE 3:**
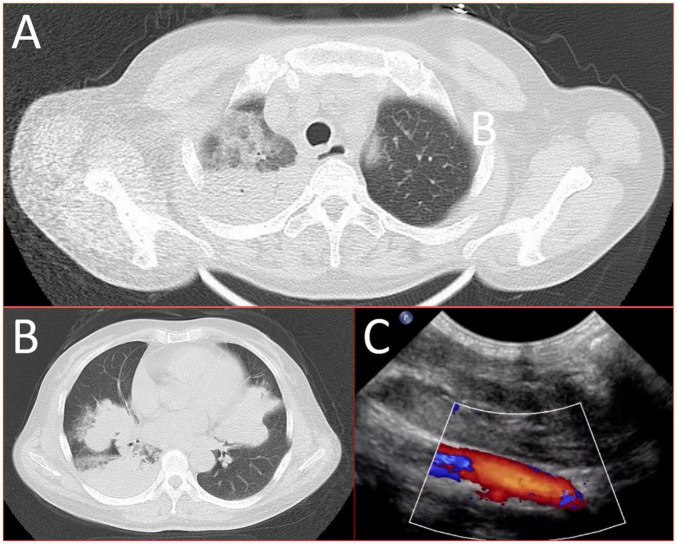
16-slice computerized tomography images of the chest in the exacerbation stage of the patient. **(A)** Upper lobe; **(B)** lower lobe and ultrasound images of the left internal jugular vein at the exacerbation stage. **(C)** The continuity of the external wall of the left internal jugular vein root was interrupted, the internal diameter of the left internal jugular vein root was approximately 3.2 mm, and the internal diameter of the upper segment was approximately 7.0 mm. The sound transmission in the middle and lower segments of the vein was poor, and faint echo filling was visible).

**FIGURE 4:**
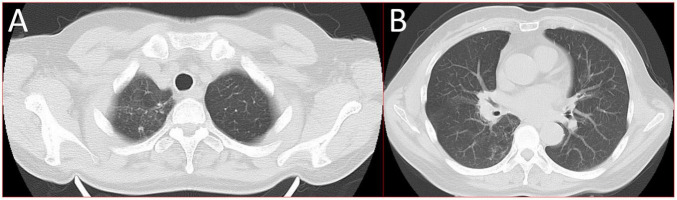
16-slice computerized tomography images of the chest taken during follow-up after the patient was cured. **(A)** Upper lobe; **(B)** lower lobe.

A 56-year-old healthy female worker was admitted to the hospital with gum pain followed by fever. Her gum pain was not obviously causing, and she did not receive any other treatment except for increasing the frequency of brushing. Two days later, the above symptoms worsened, and fever developed, with a maximum temperature of 38.7°C. A doctor found her gingival congestion and dental calculus obvious (Figure 1). The patient had no cough, no nasal congestion, no runny nose, no frequent urination, urgent urination, or urine pain. The patient reported no history of special infectious diseases, no history of pollen or pet contact, and no history of family hereditary diseases. Based on her history, she received symptomatic empirical treatment, which included tooth and oral cleaning and empirical oral metronidazole (500 mg/time, 3 times/day). Three days later, the patient had no obvious relief of the above symptoms and developed neck and pharyngeal pain and cough. On physical examination, her body temperature was 38.4°C, her pulse rate was 112 breaths/min, and her respiration rate was 20 breaths/min. Her blood pressure was 153/92 mmHg. The patient was found to have shortness of breath, and scattered moist rales could be heard in both lungs. The patient underwent routine blood and blood biochemical tests, as shown in Table 1. In addition, she underwent a chest computerized tomography (CT) examination, which revealed multiple patchy shadows in the right lung, and lymph nodes were visible in the mediastinum (Figures 2A, B). The patient also underwent an ultrasound examination of the neck, which revealed local effusion and inflammatory changes (Figures 2C, D). After discussion among the multidisciplinary team (MDT) based on her medical history, her diagnosis was first considered pulmonary infection, and the possibility of fungal infection could not be excluded. Therefore, the G test, GM test and Cryptococcus detection were performed. Moreover, voriconazole (the first two doses were 400 mg every 12 h and the maintenance dose was 200 mg for 12 h) antifungal therapy was added to the treatment mixture. Subsequent laboratory test results, including the G test, GM test and Cryptococcus antigen test, were negative. After 3 days, the patient’s symptoms progressed further. Physical examination revealed that he was conscious and wheezing and had tenderness in the right neck, a small number of moist rales audible in both lungs, a heart rate of 112 beats/min, an irregular rhythm, audible and premature beat sounds, and no murmur at cardiac auscultation. The abdomen was flat and soft; the liver, spleen and ribs were not palpated; and the lower limbs were not edematous. She received intravenous infusion of cefazolin sodium combined with intravenous infusion of metronidazole, oxygen inhalation and symptomatic treatment. Bacterial cultures of sputum and blood were performed, and no positive findings were found. During this time, the patient was re-examined by chest CT, which revealed that the patchy shadows in the right lung increased significantly, and similar patchy shadows were observed in the left lung and pericardial effusion (Figures 3A, B). Reexamination via color ultrasound of the neck revealed that the area of pus cavities in the neck increased and that there was a bacterial embolus in the jugular vein (Figure 3C). The surgeon performed pharynx and cervical incisions and drainage. The pus was milky white and was examined for pathogenic bacteria by metagenomic next-generation sequencing (mNGS), which revealed a large number of *Prevotella oris*. At this point, the patient was diagnosed with rapidly progressing infection caused by *Prevotella oris*. According to the mNGS testing result, the antibiotic was replaced by intvenous meropenem (1.0 g/time, 1 times/8 h) combined with moxifloxacin (400 mg/day) for intravenous therapy, while supportive therapy such as gammaglobulin and albumin was used. Two days later, the patient’s temperature returned to normal, and his symptoms improved significantly. After another week of treatment, the neck drain was removed, and the patient had no respiratory symptoms. The patient was subsequently discharged and continued oral moxifloxacin (400 mg/day) treatment for 2 weeks without any recurring symptoms. Three months after discharge, the patient’s symptoms did not resolve, and reexamination via computerized tomography (CT) confirmed that the patchy shadows in both lungs were basically absorbed (Figure 4).

**TABLE 1:** Patient laboratory results.

Laboratory tests	Normal range	Results
WBC (×109/L)	4∼10	16.24
Neutrophile granulocyte percentage (%)	50∼70	91.1
Eosinophils percentage (%)	0∼5	0.6
Monocytes percentage (%)	3∼8	2.3
C-reactive protein (mg/L)	<22	183
Erythrocyte sedimentation rate (mm/hour)	0∼10	26
Albumin(g/L)	35∼55	30.4
Alanine aminotransferase (U/L)	7∼40	28
Aspartate aminotransferase (U/L)	13∼35	22
Creatinine (umol/L)	41∼81	48.3
Urea nitrogen (umol/L)	3.6∼9.5	6.21
Direct bilirubin (umol/L)	0∼6.8	5.2
Procalcitonin (ng/mL)	<0.05	26.52
Interleukin-6 (pg/mL)	0∼40	>5000
*Mycoplasma pneumoniae* antibody	Negative	Negative
Chlamydia pneumoniae antibody	Negative	Negative
T-SPOT-TB	Negative	Negative
Protein (urinalysis routine)	Negative	Negative
Urine glucose (urinalysis routine)	Negative	Negative
Urobilinogen (urinalysis routine)	Negative	Negative
Occult blood (urinalysis routine)	Negative	Negative

## Discussion

*Prevotella* was first isolated from a patient with mild periodontitis by Holdeman and her/his colleagues in 1982 (10). The genus Bacteroides was reclassified as *Prevotella oris* by Shah and Collins in 1990 (11). Currently, more than 50 species have been identified in the genus *Prevotella*, occurring in diverse natural habitats, although most of these species are associated with humans (12). These *Prevotella* species implanted in the human body are widely distributed in the human microbiome and play a key role in the balance between health and disease (13). *Prevotella* is a genus of *Bacteroides* that has natural antibiotic resistance genes that bind to or attach to bacteria outside of epithelial cells, preventing their elimination (14). It mainly exists on the surface of the mucosa and has been isolated from the oral cavity and gastrointestinal and urogenital tract mucosa (15). Cases of oropharyngeal infections leading to severe pneumonia caused by *Prevotella oris* have not been reported in previous studies.

In this case, the patient developed symptoms in both lungs. The possible mechanism is local abscess formation by oropharyngeal infection in the early stage of the disease. The infection developed into pus, and the pus infected the mediastinum and lungs under the influence of gravity through the three layers of the oropharyngeal cervical fascia and the space between the submandibular, parapharyngeal or/and retropharyngeal regions. This case is worth reporting and reviewing, mainly for the following reasons.

First, serious infections can rapidly develop from common symptoms in an era of widespread antibiotic use. There is an old saying in China that illness comes from the mouth. Oropharyngeal diseases can spread to neighboring tissues and organs in a variety of ways. Oropharyngeal infections can easily invade through potential spaces and fascial planes. Without prompt treatment, the infection can spread rapidly, and complications can lead to death, making it a serious health issue (16).

Second, normally colonized *Prevotella oris* could also cause fatal pneumonia as an opportunistic pathogen (17, 18). The oral microbiome is one of the most diverse microbiomes in the human body, with more than 700 species identified (9). Oropharyngeal infections are usually polymicrobial in nature and include *Streptococci*, *Fusobacterium necrophorum*, *Bacteroides*, *Peptostreptococcus species*, *Staphylococcus aureus*, and *anaerobes* (7, 8). Immunocompetent patients rarely develop serious infections caused by *Prevotella oris* (19). Typically, these infections are caused by hematogenous spread after dental disease or manipulation. This finding suggested that *Prevotella oris* may have the ability to evade the immune system.

Third, this approach allows us to think about the standard diagnosis of such diseases. Although radiological imaging, including artificial intelligence-assisted radiological imaging, can provide more diagnostic information, there is still a certain distance from the diagnosis (20, 21). Depending on the rapid changes and poor outcomes, doctors may over diagnose these diseases. The development of diagnostic technology in recent years has made it possible to isolate and identify anaerobic bacteria in a timely manner. The cultivation of anaerobic bacteria in clinical microbiology laboratories is usually limited by strict culture environment requirements, lengthy passaging processes, loss of culture during passaging and difficulty in assessing the correlation between anaerobic bacteria and other species of flora (22). The emergence of mNGS technology allows all nucleic acid sequences present in the sample to be amplified using random primers. Ideally, all pathogens, including rare, new, or atypical pathogens responsible for complex diseases, can be detected without bias (23, 24). According to one study, mNGS reached a sensitivity of 92.31% in diagnosing pathogens (25). A further advantage of mNGS is its shorter detection time than bacterial culture (26).

## Limitations

This was a case report study, which was not highlight a potential emerging health threat, nor previously benign bacteria may become pathogenic. Only emphasized that mNGS could be used as a tool for pathogen identification.

## Conclusion

Although *Prevotella* contributes to human metabolism and health, its pathogenic properties cannot be ignored. Oropharyngeal infections caused by *Prevotella* may cause severe infections with potentially life-threatening complications. These serious infections that rapidly develop from common symptoms in an era of widespread antibiotic use not only increase patient misunderstanding but also lead to over detection and testing of such symptoms by clinicians. mNGS may be a technical tool for addressing this contradiction.

## Data Availability

The original contributions presented in this study are included in this article/supplementary material, further inquiries can be directed to the corresponding authors.
